# A randomized, double-blind, three-arm, parallel group, single-dose phase I study to evaluate the pharmacokinetic similarity between SB12 (a proposed eculizumab biosimilar) and eculizumab (Soliris) in healthy subjects 

**DOI:** 10.5414/CP204176

**Published:** 2022-03-29

**Authors:** Hyun A Lee, Hyerin Jang, Deokyoon Jeong, Younsoo Kim, Rainard Fuhr

**Affiliations:** 1 Samsung Bioepis Co., Ltd., Incheon, Republic of Korea, and; 2 Parexel International GmbH, Berlin, Germany

**Keywords:** SB12, eculizumab, biosimilar, pharmacokinetics, pharmacodynamics

## Abstract

Objectives: To compare the pharmacokinetics (PK), pharmacodynamics (PD), safety, and immunogenicity between SB12 (a proposed eculizumab biosimilar) and the reference product (RP) eculizumab (i.e., European Union (EU)-sourced Soliris and United States (US)-sourced Soliris). Materials and methods: In this phase I study, healthy adult subjects were randomized to receive a 300-mg dose of SB12 or RP eculizumab via intravenous infusion. The PK endpoints were area under the serum concentration-time curve from time zero to infinity and to the last quantifiable concentration, and maximum serum concentration. Bioequivalence for the PK endpoints was determined if the 90% confidence intervals (CIs) for the ratio of geometric least squared means (Lsmeans) were within the pre-defined bioequivalence margins of 80.00 – 125.00%. PD, safety, and immunogenicity were also investigated. Results: The 90% CIs of the geometric Lsmeans ratios of the PK endpoints were fully contained within the pre-defined bioequivalence margin. PD profiles and incidence of treatment-emergent adverse events across treatment groups were comparable. Incidence of anti-drug antibodies was also comparable between all groups, and a positive result for neutralizing antibodies was not detected. Conclusion: This study demonstrated PK bioequivalence and similar PD, safety, and immunogenicity profiles of SB12 to both reference eculizumab products.

## Introduction 

Biological medicines are originated from living cells and organisms. Monoclonal antibodies (mAbs) are biological agents that are one of the most powerful tools in modern medicine [1]. These mAbs are significantly more effective than previously approved therapies, and they are better tolerated and easier to deliver. Pipelines of mAb are rapidly growing to treat and prevent many existing and emerging infections and neglected diseases [2, 3]. However, mAbs are more complex and expensive to develop than small molecule drugs [4]. Biosimilars, which contain a version of the active ingredient of the original drug, can improve the overall health outcomes by increasing patients access to mAbs [5, 6]. While the patents for a lot of mAbs are expiring, many biosimilars are being developed. Biosimilars are assessed and compared with the original reference drug to ensure that they are highly similar and have no clinically meaningful differences in terms of structure, function, pharmacokinetics (PK), pharmacodynamics (PD), immunogenicity, clinical efficacy, and safety [6, 7, 8]. 

Eculizumab is a humanized monoclonal immunoglobulin G2/4 (IgG2/4) κ antibody [9]. It binds to the terminal complement protein C5, which acts as a late stage in the complement cascade, inhibits the complement pathway by preventing the cleavage of C5 to C5a and C5b, and thereby reduces hemolysis and thrombotic risk [10, 11]. Eculizumab is currently indicated for the treatment of paroxysmal nocturnal hemoglobinuria (PNH), atypical hemolytic uremic syndrome (aHUS), generalized myasthenia gravis, and neuromyelitis optica spectrum disorder [12, 13, 14, 15, 16, 17, 18, 19]. 

SB12 has been developed as a proposed biosimilar of Soliris (Alexion Pharmaceuticals, Inc., Boston, MA, USA) having eculizumab as the active substance; and SB12 and Soliris have an identical primary structure (data not shown). SB12 is produced by recombinant DNA technology in a Chinese hamster ovary mammalian cell expression system. A series of in vitro studies were performed to examine the similarity between SB12 and Soliris, and the results were not significantly different (data not shown). As outlined in the “Guideline on similar biological medicinal products containing monoclonal antibodies – non-clinical and clinical issues (2012)”, a risk-based approach was taken to the non-clinical evaluation of SB12 [20]. Based on the in vitro results, it was expected that SB12 will exhibit similar clinical profiles (PK, PD, safety, and immunogenicity) compared with Soliris. For phase I study, SB12 has the same dosage form and strength as the reference product Soliris. 

The primary objectives of this phase I study were to evaluate PK similarity of SB12, European Union (EU)-sourced Soliris, and United States (US)-sourced Soliris in healthy adult subjects. The secondary objectives were to evaluate the PD, safety, tolerability, and immunogenicity of SB12 compared with reference product (RP) eculizumab (EU-sourced Soliris and US-sourced Soliris) in healthy subjects. 

## Materials and methods 

### Study subjects 

Subjects included in this study were healthy females of non-childbearing potential and healthy males, both aged 18 – 55 years with body mass index (BMI) of 20.0 – 29.9 kg/m^2^ and body weight of 70.0 – 95.0 kg. Subjects were required to have normal physical examination, clinical laboratory test values, urinalysis values, vital signs, 12-lead electrocardiogram (12-lead ECG) before randomization. Key exclusion criteria were that subjects had a history or presence of clinically significant atopic allergy, allergic/hypersensitivity reactions, or known or suspected clinically relevant drug hypersensitivity to eculizumab or its excipients. Subjects with known or suspected hereditary or acquired complement deficiency were also excluded. Subjects were excluded if they had received treatment with mAb or fusion protein within 9 months prior to investigational product (IP) administration and/or showed evidence of immunogenicity from previous exposure to an mAb or fusion protein, and they had a history of invasive systemic fungal infections or other opportunistic infections. Subjects with a positive test for hepatitis B surface antigen, hepatitis B core antibody, hepatitis C virus antibody, or human immunodeficiency virus (HIV) were excluded. Potential eculizumab side effects were mitigated by specific inclusion criteria and restrictions, and all participants were vaccinated against *Neisseria meningitidis* with available vaccines against the most common local strains. 

### Study design 

This was a randomized, double-blind, three-arm, parallel group, single-dose study in healthy male and female subjects. This study was performed at a single center in Germany from November 2018 to April 2019 (clinicaltrials.gov identifier: NCT03722329; EudraCT number: 2018-001858-10). 

A total of 240 subjects were randomized in a ratio of 1 : 1 : 1 to receive a 300-mg single dose of either SB12 or RP eculizumab via intravenous (IV) infusion over 35 minutes. Subjects were discharged on day 3 after study procedures were completed. They returned to the clinical research unit on an outpatient basis on days 5, 8, 11, 15, 22, 29, 36, 43, 50, 57, and 64 (end of study (EOS) visit) for PK, PD, safety, and immunogenicity assessments. 

The final study protocol was approved by the responsible local Independent Ethics Committee (IEC) in Germany. This study was conducted in accordance with the ethical principles that have their origin in the Declaration of Helsinki (1996) and that are consistent with the latest International Council for Harmonisation (ICH) E6 (R2) Good Clinical Practice (GCP) guideline and applicable local regulatory requirements and laws in Germany. The informed consent documents for this study were approved by the IEC prior to use. 

### Pharmacokinetic evaluation 

Blood samples (~ 4.0 mL) for PK analysis of serum eculizumab concentrations (SB12 or RP eculizumab) were collected at 0 (pre-dose), 0.58 (end of infusion), 4, 8, 12, 24, and 48 hours, then at day 5 (96 hours), 8 (168 hours), 11 (240 hours), 15 (336 hours), 22 (504 hours), 29 (672 hours), 36 (840 hours), 43 (1,008 hours), 50 (1,176 hours), 57 (1,344 hours), and 64 (1,512 hours) after start of infusion. Collected blood samples were clotted for 1 – 2 hours, and then centrifuged at 2,500 – 3,000 g for ~ 10 minutes at 4 °C. The serum was stored at –80 °C or below until analysis. PK samples were analyzed by a qualified laboratory, and the serum concentration of eculizumab was measured using the validated electrochemiluminescent format with acid dissociation specific for the detection and quantification of eculizumab in human serum. SB12 was used to prepare calibration standard samples and quality control (QC) samples. All samples underwent acid dissociation to release any endogenous C5 protein bound with SB12. Samples were then neutralized and incubated with capture solution to allow SB12 to bind to excess biotinylated C5. After incubation with excess biotinylated C5, samples were added to the streptavidin-coated MSD plate, following incubation with sulfo-TAG labelled anti-human IgG4 antibody. This assay visualized by the additions of read buffer containing tripropylamine, which produced a chemiluminescent signal when an electrical voltage was applied. Inter-day precision (percent coefficients variation (%CV)) for the QC samples was 9.2 – 12.6%, and the accuracy (%bias) ranged from –4.8 to 3.5%. The lower limit of quantification (LLOQ) and upper limit of quantification (ULOQ) was 0.8 and 12.5 μg/mL, respectively. 

The primary PK endpoint was area under the serum concentration-time curve (AUC) from time zero to infinity (AUC_inf_). The secondary PK endpoints were AUC from time zero to the last quantifiable serum concentration (AUC_last_), maximum serum concentration (C_max_), time to reach C_max_ (t_max_), volume of distribution during terminal phase (V_z_), terminal rate constant (λ_z_) calculated by linear least squares regression analysis using the last 3 (or more) non-zero serum concentrations, terminal half-life (T_1/2_) calculated by ln(2)/λ_z_, total body clearance, and percentage of extrapolated AUC from last quantifiable serum concentration to infinity (%AUC_extrap_). 

### Pharmacodynamic evaluation 

Blood samples (~ 9.5 mL for pre-dose and 8.5 mL for post-dose) for assessment of the terminal complement activity were collected at 0 (pre-dose), 0.58 (end of infusion), 4, 24, 48 hours, then at day 5 (96 hours), 8 (168 hours), 11 (240 hours), 15 (336 hours), and 64 (1,512 hours) after start of infusion. Collected blood samples were clotted for 1 – 2 hours, and then centrifuged at 2,500 – 3,000 g for ~ 10 minutes at 4 °C. The serum was stored at –80 °C or below until analysis. 

The PD endpoint was change in terminal complement activity over time. PD samples were analyzed by the qualified laboratory. The wieslab enzyme-linked immunosorbent assay (ELISA) combines principles of the hemolytic assay for complement activation with the use of alkaline phosphatase (APh)-conjugated antibodies specific for the neoantigen produced as a result of complement activation [21]. The amount of neoepitope generated is proportional to the functional activity of the complement pathway. Calibration standards are prepared using a reconstituted positive control standard provided as part of a wieslab ELISA kit. Because the wells are coated with specific activators, such as human IgM, the classical complement pathway in human serum is activated during serum incubation at 37 °C. The amount of terminal complement complex (TCC) formed on the plate surface is detected by a specific APh-conjugated antibody to the neoepitope of C5b-9. The percentage of terminal complement activity in human serum samples is then back-calculated from the calibration standard curve. The LLOQ and ULOQ was 10% and 125%, respectively. 

### Safety evaluations 

The safety endpoints were adverse events (AEs) and serious AEs (SAEs), clinical laboratory values (hematology, biochemistry, and urinalysis), 12-lead ECG, vital signs, and physical examination. 

All AEs including SAEs that occurred from screening (days –28 to –2) to the EOS (day 64) were recorded. The AEs that emerged during treatment with IP (i.e., treatment-emergent AE (TEAE)) and AEs of special interest (AESIs) were analyzed for the purposes of safety analysis. Meningococcal infections, other systemic infections, and infusion-related reactions were classified as AESI. 

All AEs were coded according to the Medical Dictionary for Regulatory Activities (MedDRA) version 21.0, and listed separately by treatment group including subject number, preferred term, seriousness, and severity. The AEs were classified as mild, moderate, or severe. The severity assessment was supported and guided by the grading of the National Cancer Institute-Common Toxicity Criteria for AEs (NCI-CTCAE) version 5.0 to describe the maximum intensity of the AE wherever reasonable in healthy subjects. 

### Immunogenicity evaluations 

Blood samples (~ 11.0 mL) for assessment of immunogenicity (i.e., incidence of anti-drug antibodies (ADAs) and neutralizing antibodies (NAbs)) were collected at day 1 (0 hours, pre-dose), 15 (336 hours), 29 (672 hours), and 64 (1,512 hours) after start of infusion. 

Immunogenicity samples were analyzed at a qualified laboratory using validated methods with a multi-tiered approach, consisting of a screening and a confirmatory assay for ADAs as well as a titer and a neutralization assay. Binding ADAs were detected using a qualitative and semiquantitative electrochemiluminescent bridging assay. In ADA-positive response samples, the NAbs were detected using an electrochemiluminescent competitive ligand assay. A subject was considered to be ADA-positive if both screening and confirmatory testing were positive. Post-dose ADA-positive was defined as subjects with at least 1 ADA positive post-baseline (i.e., day 15, 29, or 64), and post-dose ADA-negative was defined as subjects without any ADA-positive post-baseline. Post-dose NAb was defined as positive if the subject had at least 1 positive Nab result post-baseline. 

### Statistical analysis 

The sample size was calculated based on an estimated inter-subject %CV of 42%, which was calculated from that reported in previously published data [9]. With a sample size of 72 in each of the 3 treatment groups (i.e., a total sample size of 216), a parallel design would have 90% power assuming a true geometric mean ratio of 1.00 to be able to reject both the null hypotheses that 1) the true geometric mean ratio of the test to the reference is less than 80%, and 2) the true geometric mean ratio of the test to the reference is larger than 125%, where both of these null hypotheses could be rejected simultaneously if the 90% confidence intervals (CIs) for the true geometric mean ratio lies completely between 80 and 125%. 

Assuming a 10% drop-out rate, a total of ~ 240 subjects (80 subjects in each treatment groups) were enrolled for this study. 

The safety set included all subjects who received the IPs and were used for the analysis of safety and immunogenicity. PK and PD analysis were performed in the PK analysis set, which included all subjects in the safety set without any major protocol deviation. 

Equivalence for the primary (i.e., AUC_inf_) and secondary PK endpoints (i.e., AUC_last_ and C_max_) was determined if the 90% CIs for the ratio of geometric least squared means (Lsmeans) of SB12 to EU-sourced Soliris, SB12 to US-sourced Soliris, and EU-sourced Soliris to US-sourced Soliris was within the pre-defined bioequivalence margins of 80.00 – 125.00%. The statistical analysis of the log_e_-transformed PK endpoints was performed by an analysis of variance (ANOVA) model with treatment group as a fixed effect. The difference in LSMeans between SB12 and EU-sourced Soliris, between SB12 and US-sourced Soliris, or between EU-sourced Soliris and US-sourced Soliris and corresponding 90% CIs were determined. Back-transformation provided the ratio of geometric Lsmeans and 90% CIs for these ratios. PK parameters for analysis were calculated based on actual sampling times and a non-compartmental analysis method using Phoenix WinNonlin (Certara, St. Louis, MO, USA) version 8.0. 

There were no statistical comparisons between the treatment groups for PD data. The change from baseline in terminal complement activity was calculates as follows: 

Change in terminal complement activity (%) = (complement activity at each timepoint – complement activity at baseline)/(complement activity at baseline) × 100 

## Results 

### Study subjects 

**Figure 1.**
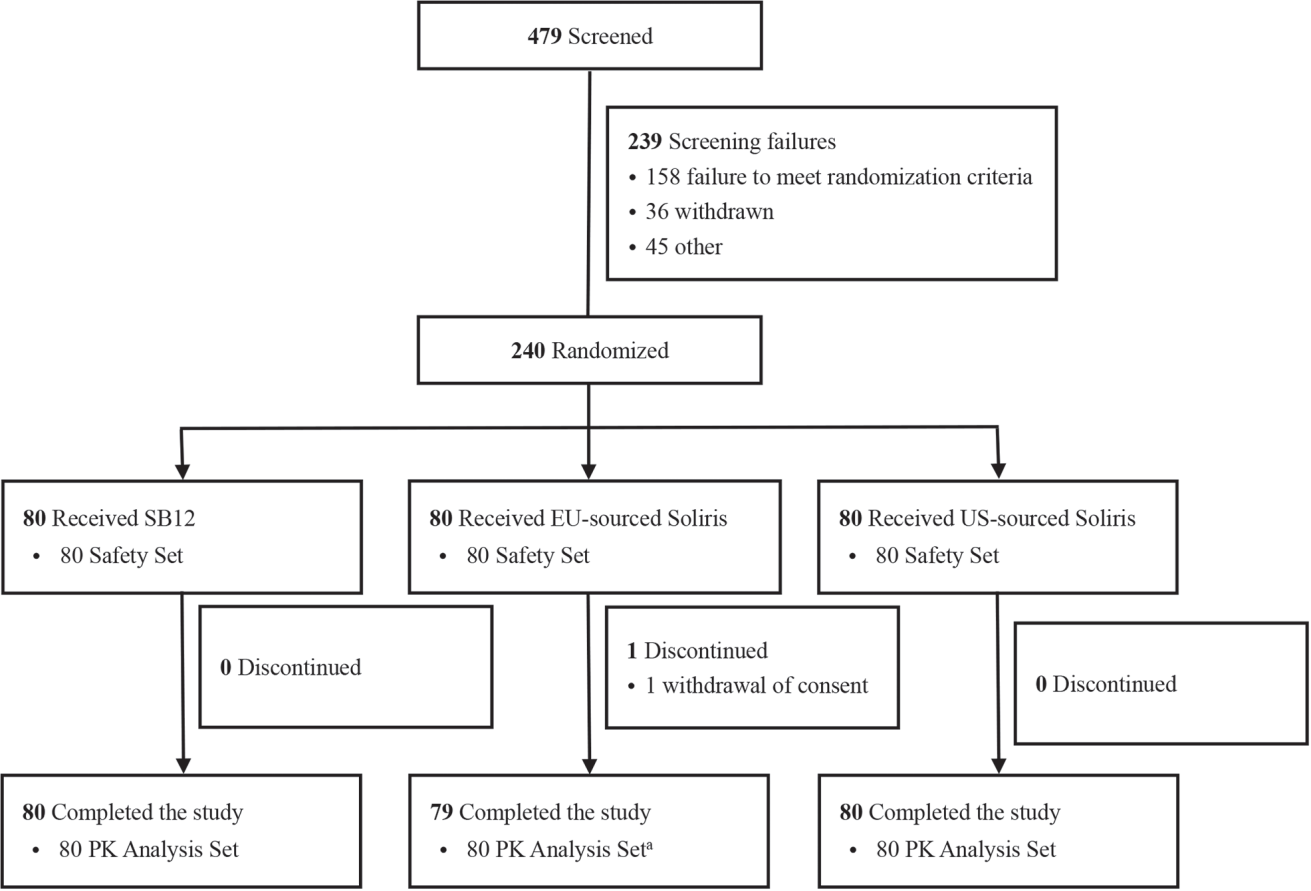
CONSORT diagram of participants’ flow through the trial. EU-sourced Soliris = European Union-sourced eculizumab; PK = pharmacokinetic; US-sourced Soliris = United States-sourced eculizumab. ^a^One subject in EU-sourced Soliris treatment group discontinued the study due to withdrawal of informed consent, and this subject was excluded from primary PK analysis.

A total of 240 subjects were randomized and received a single dose of study drug (SB12, n = 80; EU-sourced Soliris, n = 80; US-sourced Soliris, n = 80), of which 239 completed the study (Figure 1). One subject (0.4%) in the EU-sourced Soliris treatment group discontinued the study due to withdrawal of informed consent, and this subject was excluded from primary PK analysis. None of the subjects discontinued the study due to an AE. All subjects were included for the PK analysis set because no subject had major protocol deviations. 230 subjects (95.8%) were male, and 10 subjects (4.2%) were female in this study. The majority of subjects were White (230 subjects, 95.8%) and not Hispanic or Latino (236 subjects, 98.3%) (Table 1). Demographics and other baseline characteristics were generally comparable between the treatment groups (Table 1). 

### Pharmacokinetics and pharmacodynamics 

**Figure 2.**
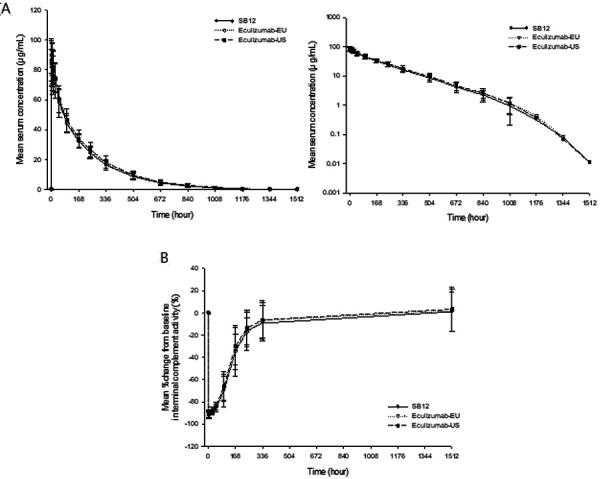
A: Mean (± SD) serum SB12, EU-sourced Soliris, and US-sourced Soliris concentration-time profiles; Linear (left) and semi-logarithmic (right) scales. B: Mean (± SD) change from baseline in terminal complement activity of SB12, EU-sourced Soliris, and US-sourced Soliris. EU-sourced Soliris = European Union-sourced eculizumab; SD = standard deviation; US-sourced Soliris = United States-sourced eculizumab.

The mean serum concentration-time profiles between treatment groups (i.e., SB12, EU-sourced Soliris, and US-sourced Soliris) were superimposable (Figure 2). PK parameters including t_max_ and T_1/2_ were also comparable across the treatment groups. The median of maximum serum concentrations was reached between 0.7 and 4.0 hours after the start of infusion, following which, concentrations declined in a biphasic manner, and the mean of terminal T_1/2_ was 176.7 – 183.9 hours (Table 2). 

For all comparisons (SB12 vs. EU-sourced Soliris, SB12 vs. US-sourced Soliris, and EU-sourced Soliris vs. US-sourced Soliris), the 90% CIs of the geometric Lsmeans ratios of the primary (AUC_inf_) and secondary PK endpoints (C_max_ and AUC_last_) were fully contained within the pre-defined bioequivalence margin of 80.00 – 125.00%, thus establishing PK similarity (Table 3). 

The mean change from baseline in terminal complement activity-time profiles between treatment groups (i.e., SB12, EU-sourced Soliris, and US-sourced Soliris) were similar and overlapped (Figure 2). 

### Safety 

A total of 427 TEAEs were reported in 165 (68.8%) subjects. 149 TEAEs were reported in 56 (70.0%) subjects in the SB12 treatment group, 125 TEAEs were reported in 52 (65.0%) subjects in the EU-sourced Soliris treatment group, and 153 TEAEs were reported in 57 (71.3%) subjects in the US-sourced Soliris treatment group (Table 4). The majority of TEAEs were mild to moderate in severity. A total of 3 (1.3%) of the subjects experienced severe TEAEs (i.e., 1 subject in each of the SB12, EU-sourced Soliris, and US-sourced Soliris treatment groups) that were not related to the study drug. The proportion of subjects who experienced TEAEs considered to be related to the IPs were 31.3% of the subjects in the SB12 treatment group, 21.3% of the subjects in the EU-sourced Soliris treatment group, and 36.3% of the subjects in the US-sourced Soliris treatment group. The most frequently reported TEAE suspected to be IP-related was headache (12.5% of subjects in the SB12 treatment group, 6.3% of subjects in the EU-sourced Soliris treatment group, and 10.0% of subjects in the US-sourced Soliris treatment group). Also, the most frequently reported AESI was infusion-related reactions (IRRs) (7.5% of subjects in the SB12 treatment group, 5.0% of subjects in the EU-sourced Soliris treatment group, and 1.3% of subjects in the US-sourced Soliris treatment group). There were no deaths or discontinuations due to TEAEs and AESIs during the study. Two SAEs were reported in 1 subject in each of the SB12 and US-sourced Soliris treatment groups. A case of back pain occurred on the day of administration of the IP in the US-sourced Soliris treatment group, resolved after a duration of 4 days, and was considered as not related to the IP. Another case of renal colic occurred at 23 days after the administration of IP in the SB12 treatment group, resolved after a duration of 5 days, and was considered as not related to the IP. Clinical laboratory data, vital signs, and 12-lead ECG parameters did not show any clinically relevant changes over time that might be considered related to the study drugs. 

### Immunogenicity 

The post-dose incidence of subjects with post-dose ADA-positive to eculizumab was 2 (2.5%), 1 (1.3%), and 0 (0.0%) subjects in the SB12, EU-sourced Soliris, and US-sourced Soliris treatment groups, respectively. The incidence of ADA to eculizumab was comparable across all 3 treatment groups, and there was no statistically significant difference in the incidence of ADA between SB12 and EU-sourced Soliris (p-value = 1.00), SB12 and US-sourced Soliris (p-value = 0.50), and EU-sourced Soliris and US-sourced Soliris (p-value = 0.50). None of the subjects with post-dose ADA to eculizumab had a positive result for NAb. 

## Discussion 

SB12 is being developed as a proposed biosimilar to Soliris. The results of this phase I study demonstrate bioequivalence of SB12 to reference products, EU-sourced Soliris and US-sourced Soliris, for primary and secondary PK endpoints. The 90% CIs for the test-to-reference ratios of the calculated PK parameters (AUC_inf_, AUC_last_, and C_max_) were within the pre-defined bioequivalence criteria of 80.00 – 125.00% for the comparison of SB12 to EU-sourced Soliris and US-sourced Soliris; and of EU-sourced Soliris to US-sourced Soliris, demonstrating PK similarity among the 3 treatment groups. According to EMA and FDA requirements for assessment of similarity in PK, the study results confirm that SB12 and EU-sourced Soliris, SB12 and US-sourced Soliris, and EU-sourced Soliris and US-sourced Soliris are bioequivalent. Pharmacodynamic profiles in terms of change from baseline in terminal complement activity were comparable following administration of SB12, EU-sourced Soliris, and US-sourced Soliris. 

Safety profiles were also comparable between the treatment groups. No typical adverse reaction attributed to eculizumab or its mode of action was observed in the single-dose study. The development of binding ADAs was comparable between all treatment groups, and no NAbs were detected. The results of PK bioequivalence using ADA-negative post-dose subjects were similar to those obtained in the primary PK analysis. PK bioequivalence analyses were not performed in the ADA-positive post-dose subjects due to the very small subject numbers. Overall, these results confirmed that there were no differences in immunogenicity profiles between treatment groups (SB12, EU-sourced Soliris, and US-sourced Soliris) although the ADA-positive post-dose subject numbers were very small. 

The PD endpoint, terminal complement activity, was selected based on the correlation with clinical outcome and the understanding of mechanism of action (MOA) in PNH patients. The pathophysiology of PNH is directly linked to complement-mediated destruction of the red blood cells, which result in intravascular hemolysis, the primary clinical indication in all PNH patients, which relates to mortality in these patients. Thus, the measurement of the complement activity is an appropriated endpoint for eculizumab. The PD profile of SB12 was similar to known eculizumab profiles [22]. There was a rapid decrease in the complement activity at the end of infusion and then a slow restoration. There was no non-responder in the aspect of the measured complement activity after treatment. 

In this phase I study, the lowest therapeutic dose (300 mg) of reference product indicated for aHUS patients with a body weight ranging from 10 kg to less than 20 kg was used to reduce the possible AE risks to healthy subjects and to obtain sufficient PK for demonstration of similarity and PD for evaluation of similarity [14]. Also, at the 300-mg dose used in the biosimilar study of ABP959 (Amgen Inc., Thousand Oaks, CA, USA), PK and PD similarity between ABP959 and eculizumab were demonstrated [22]. Based on the information, a single IV infusion of 300 mg eculizumab for healthy subjects was reasonable to show PK similarity between SB12 and Soliris. 

## Conclusion 

The present study demonstrates the PK similarity of SB12 to both EU-sourced Soliris and US-sourced Soliris, and of EU-sourced Soliris to US-sourced Soliris in healthy subjects. All three study drugs also showed comparable PD, safety, and immunogenicity profiles. These results represent an important contribution to the totality of evidence for supporting the biosimilarity of SB12 to its reference drugs, following the demonstration of analytical and functional similarity in extensive quality and non-clinical results. This totality of evidence will be complemented by further clinical phase III study in patients with PNH, demonstrating bioequivalence of SB12 and eculizumab in terms of efficacy (lactate dehydrogenase (LDH) level), PD (terminal complement activity), PK, safety, and immunogenicity. 

## Authors' contributions 

H. Lee and H. Jang were responsible for manuscript preparation; H. Jang and D. Jeong were responsible for study concepts and design; Y. Kim was responsible for data analysis and interpretation; R. Fuhr was responsible for data acquisition; H. Lee, H. Jang, D. Jeong, Y. Kim, and R. Fuhr were responsible for manuscript review and approval. 

## Trial registration 

Clinicaltrials.gov identifier: NCT03722329; EudraCT number: 2018-001858-10. 

## Funding 

This study was funded by Samsung Bioepis Co., Ltd., Incheon, Republic of Korea. 

## Conflict of interest 

All authors are employees of either Samsung Bioepis Co., Ltd. or of an organization contracted by Samsung Bioepis Co., Ltd. for the present study. There are no other relationships or activities that could appear to influence the submitted work. 

**Table 1.** Demographics and other baseline characteristics (randomized set).

	SB12 (N = 80)	EU-sourced Soliris (N = 80)	US-sourced Soliris (N = 80)	Total (N = 240)
Age (years)	39.6 ± 10.7	40.9 ± 9.3	40.2 ± 9.2	40.2 ± 9.7
Gender, n (%)^a^				
Male	78 (97.5)	76 (95.0)	76 (95.0)	230 (95.8)
Female	2 (2.5)	4 (5.0)	4 (5.0)	10 (4.2)
Race, n (%)^a,b^				
White	79 (98.8)	75 (93.8)	76 (95.0)	230 (95.8)
Black or African American	0 (0.0)	1 (1.3)	0 (0.0)	1 (0.4)
Asian	0 (0.0)	1 (1.3)	0 (0.0)	1 (0.4)
American Indian or Alaska Native	0 (0.0)	0 (0.0)	1 (1.3)	1 (0.4)
Other	1 (1.3)	3 (3.8)	3 (3.8)	7 (2.9)
Ethnicity, n (%)^a,b^				
Hispanic or Latino	0 (0.0)	0 (0.0)	2 (2.5)	2 (0.8)
Not Hispanic or Latino	80 (100.0)	78 (97.5)	78 (97.5)	236 (98.3)
Unknown	0 (0.0)	2 (2.5)	0 (0.0)	2 (0.8)
Height (cm)	179.9 ± 6.0	180.6 ± 6.9	180.5 ± 6.5	180.3 ± 6.4
Weight (kg)	82.3 ± 6.5	81.3 ± 6.7	83.5 ± 6.8	82.4 ± 6.7
BMI (kg/m^2^)	25.5 ± 2.2	25.0 ± 2.3	25.7 ± 2.0	25.4 ± 2.2

Data are presented in mean ± SD unless otherwise indicated. BMI = body mass index; EU-sourced Soliris = European Union-sourced eculizumab; N = number of randomized subjects; n = number of subjects with available result; SD = standard deviation; US-sourced Soliris = United States-sourced eculizumab. ^a^Percentages are based on the number of randomized participants. ^b^We separately collected demographic information about race and ethnicity as per FDA guidance “Collection of Race and Ethnicity Data in Clinical Trials” (https://www.fda.gov/media/75453/download).

**Table 2.** Summary of PK parameters (PK analysis set).

Parameter	Statistics	SB12 (N = 80)	EU-sourced Soliris (N = 80)^a^	US-sourced Soliris (N = 80)
AUC_inf _ (h×μg/mL)	Mean ± SD	16,834.3 ± 2,426.8	17,008.9 ± 2,449.9	17,773.1 ± 3,138.7
	Median (Min, Max)	16,339.7 (12,046.0, 23,813.0)	16,759.5 (10,801.0, 22,665.0)	17,513.4 (11,538.0, 30,742.0)
	Geo. Mean ± Geo. SD	16,671.6 ± 1.2	16,830.3 ± 1.2	17,523.1 ± 1.2
AUC_last _ (h×μg/mL)	Mean ± SD	16,498.8 ± 2,373.3	16,709.1 ± 2,435.3	17,482.1 ± 3,128.5
	Median (Min, Max)	16,044.2 (11,835.0, 23,515.0)	16,493.3 (10,622.0, 22,382.0)	17,137.7 (11,325.0, 30,493.0)
	Geo. Mean ± Geo. SD	16,340.4 ± 1.2	16,529.3 ± 1.2	17,230.6 ± 1.2
C_max _ (μg/mL)	Mean ± SD	91.6 ± 11.7	84.3 ± 12.5	88.8 ± 12.9
	Median (Min, Max)	89.2 (69.0, 120.6)	83.4 (61.8, 120.8)	87.8 (62.1, 124.7)
	Geo. Mean ± Geo. SD	90.9 ± 1.1	83.4 ± 1.2	87.9 ± 1.2
t_max _ (hour)	Median (Min, Max)	0.7 (0.6, 24.2)	4.0 (0.6, 24.0)	2.3 (0.6, 24.2)
	Geo. Mean ± Geo. SD	1.7 ± 3.0	2.1 ± 3.4	2.1 ± 3.7
T_1/2 _ (hour)	Mean ± SD	183.9 ± 101.7	180.8 ± 25.1	176.7 ± 20.9
	Median (Min, Max)	171.9 (100.7, 1,051.5)	179.1 (97.0, 232.3)	177.1 (97.8, 247.0)
	Geo. Mean ± Geo. SD	174.7 ± 1.3	178.9 ± 1.2	175.4 ± 1.1
V_z _ (mL)	Mean ± SD	4,705.7 ± 2,030.7	4,656.7 ± 718.3	4,381.8 ± 658.3
	Median (Min, Max)	4,625.4 (3,042.0, 21,832.0)	4,514.7 (2,799.0, 6,337.0)	4,329.5 (2,722.0, 6,220.0)
	Geo. Mean ± Geo. SD	4,535.9 ± 1.3	4,601.3 ± 1.2	4,332.0 ± 1.2
CL (mL/hour)	Mean ± SD	18.2 ± 2.4	18.0 ± 2.7	17.4 ± 2.8
	Median (Min, Max)	18.4 (12.6, 24.9)	17.9 (13.2, 27.8)	17.1 (9.8, 26.0)
	Geo. Mean ± Geo. SD	18.0 ± 1.1	17.8 ± 1.2	17.1 ± 1.2

^a^PK parameters except for C_max_ and t_max_ in EU-sourced Soliris treatment group were calculated from 79 subjects due to early termination of 1 subject. AUC_inf_ = area under the concentration-time curve from time zero to infinity; AUC_last_ = area under the concentration-time curve from time zero to the last quantifiable concentration; CL = total body clearance; C_max_ = maximum observed serum concentration; EU-sourced Soliris = European Union-sourced eculizumab; Geo. Mean = geometric least square mean; Geo. SD = geometric standard deviation; N = number of participants in the PK analysis set; SD = standard deviation; t_max_ = time at which C_max_ was observed; T_1/2_ = terminal half-life; US-sourced Soliris = United States-sourced eculizumab; V_z_ = volume of distribution during the terminal phase.

**Table 3.** Statistical comparison of primary PK parameters between test and reference products (PK analysis set).

Test	Reference	PK parameter	n		Geometric Lsmean		Ratio (%) (90% CI)
			Test	Reference	Test	Reference	
SB12 (N = 80)	EU-sourced Soliris (N = 80)	AUC_inf _ (h×μg/mL)	80	79	16,671.6	16,830.3	99.1 (95.41,102.85)
		AUC_last _ (h×μg/mL)	80	79	16,340.4	16,529.3	98.9 (95.19, 102.66)
		C_max _ (μg/mL)	80	80	90.881	83.437	108.9 (105.09, 112.89)
SB12 (N = 80)	US-sourced Soliris (N = 80)	AUC_inf _ (h×μg/mL)	80	80	16,671.6	17,523.1	95.1 (91.40, 99.04)
		AUC_last _ (h×μg/mL)	80	80	16,340.4	17,230.6	94.8 (91.09, 98.74)
		C_max _ (μg/mL)	80	80	90.881	87.914	103.4 (99.75, 107.13)
EU-sourced Soliris (N = 80)	US-sourced Soliris (N = 80)	AUC_inf _ (h×μg/mL)	79	80	16,830.3	17,523.1	96.0 (92.16, 100.10)
		AUC_last _ (h×μg/mL)	79	80	16,529.3	17,230.6	95.9 (92.00, 100.03)
		C_max _ (μg/mL)	80	80	83.437	87.914	94.9 (91.36, 98.59)

AUC_inf_ = area under the concentration-time curve from time zero to infinity; AUC_last_ = area under the concentration-time curve from time zero to the last quantifiable concentration; CI = confidence interval; C_max_ = maximum observed serum concentration; EU-sourced Soliris = European Union-sourced eculizumab; Lsmean = least square mean; N = number of participants in the PK analysis set; n = number of participants in the analysis; PK = pharmacokinetic; US-sourced Soliris = United States-sourced eculizumab.

**Table 4.** Summary of adverse events (safety set).

Category	SB12 (N = 80) n (%)	EU-sourced Soliris (N = 80) n (%)	US-sourced Soliris (N = 80) n (%)	Total (N = 240) n (%)
Any AE	58 (72.5)	54 (67.5)	59 (73.8)	171 (71.3)
Any TEAE	56 (70.0)	52 (65.0)	57 (71.3)	165 (68.8)
Any SAE	1 (1.3)^a^	0 (0.0)	1 (1.3)^b^	2 (0.8)
Any AESI	6 (7.5)	4 (5.0)	3 (3.8)	13 (5.4)
Meningococcal infection	0 (0.0)	0 (0.0)	0 (0.0)	0 (0.0)
Other systemic infection	0 (0.0)	0 (0.0)	2 (2.5)	2 (0.8)
Infusion-related reaction	6 (7.5)	4 (5.0)	1 (1.3)	11 (4.6)
TEAE severity				
Mild	36 (45.0)	28 (35.0)	24 (30.0)	88 (36.7)
Moderate	19 (23.8)	23 (28.8)	32 (40.0)	74 (30.8)
Severe	1 (1.3)	1 (1.3)	1 (1.3)	3 (1.3)
TEAE causality				
Related	25 (31.3)	17 (21.3)	29 (36.3)	71 (29.6)
Not related	31 (38.8)	35 (43.8)	28 (35.0)	94 (39.2)
TEAEs occurring in ≥ 5% of participants				
Headache	17 (21.3)	12 (15.0)	19 (23.8)	48 (20.0)
Nasopharyngitis	18 (22.5)	18 (22.5)	10 (12.5)	46 (19.2)
Rhinitis	7 (8.8)	7 (8.8)	4 (5.0)	18 (7.5)
Back pain	8 (10.0)	4 (5.0)	5 (6.3)	17 (7.1)
Upper respiratory tract infection	1 (1.3)	7 (8.8)	5 (6.3)	13 (5.4)
Infusion-related reactions	6 (7.5)	4 (5.0)	1 (1.3)	11 (4.6)
Diarrhea	2 (2.5)	2 (2.5)	7 (8.8)	11 (4.6)
Nausea	4 (5.0)	1 (1.3)	4 (5.0)	9 (3.8)
Influenza-like illness	2 (2.5)	2 (2.5)	5 (6.3)	9 (3.8)
Oropharyngeal pain	2 (2.5)	4 (5.0)	3 (3.8)	9 (3.8)
Pain in extremity	2 (2.5)	1 (1.3)	5 (6.3)	8 (3.3)
Dizziness	1 (1.3)	0 (0.0)	5 (6.3)	6 (2.5)

AE = adverse event; AESI = AE of special interest; EU-sourced Soliris = European Union-sourced eculizumab; N = number of participants in the safety set; n = number of subjects with that observation; PT = preferred term; SAE = serious AE TEAE = treatment-emergent AE; US-sourced Soliris = United States-sourced eculizumab. Percentages are based on the number of participants in the safety set. If a participant had multiple events with different severity (or causality), then the participant was counted only once at the worst severity (or causal ity). ^a^Renal colic in SB12 group; ^b^back pain in US-sourced Soliris group.
